# Drug therapy problems and associated factors among adult patients admitted to the surgical ward in a resource-limited setting

**DOI:** 10.3389/fphar.2025.1548105

**Published:** 2025-07-04

**Authors:** Taju Seid, Jemal Abdela, Mesay Dechasa, Shambel Nigussie

**Affiliations:** ^1^ Department of Pharmacy, Clinical Pharmacy Unit, Wachemo University, Hossana, Ethiopia; ^2^ Department of Pharmacology, School of Pharmacy, College of Health and Medical Sciences, Haramaya University, Harar, Ethiopia; ^3^ Department of Clinical Pharmacy, School of Pharmacy, College of Health and Medical Sciences, Haramaya University, Harar, Ethiopia; ^4^ Discipline of Pharmacy, University of Canberra, Canberra, ACT, Australia

**Keywords:** drug therapy problems, magnitude, prevalence, surgical patients, central Ethiopia

## Abstract

**Background:**

Drug therapy problems are common in hospitalized patients and may lead to increased hospital stays, healthcare costs, and increased risk of morbidity and mortality. Despite these facts, limited data exist on the magnitude of DTPs and associated factors among adult surgical patients in resource-limited settings, particularly in a current study setting. Therefore, this study aimed to assess the magnitude of DTPs and associated factors among adult surgical patients at study setting in Ethiopia.

**Methods:**

A hospital-based cross-sectional study was conducted at Wachemo University Comprehensive Specialized Hospital from January 1 to 30 March 2024. Data were collected through patients’ interviews and chart reviews using pre-tested questionnaires and data abstraction formats. Data were analyzed using Statistical Package for Social Science version 20. Factors associated with drug therapy problems were determined by binary logistic regression analysis. A p-value of less than or equal to 0.05 was considered statistically significant in the final analysis, and an adjusted odds ratio with a 95% confidence interval was used to determine the strength of the association.

**Results:**

The total number of recruited patients was 330. Of them, 304 participants who fulfilled the inclusion criteria. Of those participants, 216 (71.1%) were males. The mean age of the study participants was 44 (±17) years. Nearly half of the patients encountered at least one drug therapy problem, and 464 drug therapy problems were identified with a magnitude of 73.68% (95% CI: 0.684–0.785). Non-compliance (27.0%) was the most frequently identified drug therapy problem, followed by a need for additional drug therapy (21.1%). Length of hospital stay ≥7 days [adjusted odds ratio (AOR) = 2.47 [95% confidence interval (CI) 1.243–4.909, p = 0.01]), ≥5 drugs taken per day (AOR = 2.874 [95%CI: 1.411–5.851, p = 0.004]), and postoperative antibiotic use (AOR = 0.057 [95%CI: [0.028–0.115, p = 0.001]) significantly affect drug therapy problems.

**Conclusion:**

This study identified a high prevalence of DTPs that was independently predicted by the presence of polypharmacy, prolonged hospital stay (≥7 days), and postoperative antibiotic use. Non-compliance were the most frequent identified drug therapy problems. Therefore, early identification of drug therapy problems and the associated factors may enhance the prevention and management of drug therapy problems.

## Introduction

Drugs are vital tools in healthcare services that contribute to the improvement in the quality and expectancy of patients’ lives by preventing, and curing diseases (Muche et al., 2022). However, when drugs are used improperly, they can lead to drug-related problems (Patil et al., 2019). Drug therapy problems (DTPs) refer to a situation concerning pharmaceutical care practice that either directly or indirectly hinders the desired treatment outcomes (Abate et al., 2020; Tegegn et al., 2018; Cipolle et al., 2012a). The World Health Organization (WHO) launched “The Global Patient Safety Challenge: Medication without Harm” to stop preventable pharmaceutical damage. A recognized technique for classifying DTPs in a variety of contexts is the Pharmaceutical Care Network Europe (PCNE) categorization system, which may be incorporated into routine pharmaceutical care operations (Organization, 2024). DTPs are a significant contributor to morbidity and mortality and create considerable pressure on the healthcare systems (Alayed et al., 2019). It is estimated that approximately 5%–10% of all hospital admissions are drug-related, and about 22% of patients are discharged with DTPs. As many as 28% of all emergency department visits are drug-related issues (Belayneh et al., 2018).

Around 50% of patients undergoing surgery regularly take a significant number of medications both for surgery and for reasons unrelated to surgery, putting them at risk for drug problems and postoperative complications (Mohammed et al., 2022). As a result, it is important to identify and address DTPs as soon as possible (Tefera et al., 2020). Globally, fewer studies directly assessed DTP among surgical patients, and the majority of the studies focused on the issue of antibiotic misuse and their costs, adverse drug events, and medication errors (Krähenbühl-Melcher et al., 2007; Mejía et al., 2020; Niriayo et al., 2023). However, patients in the surgical ward also utilize different drug classes for various illness conditions in addition to antimicrobials, which require judicious administration for better results (Mattana et al., 2024).

The prevalence and factors associated with DTPs vary in diverse studies. For example, DTPs were generally documented by several studies in various nations, regardless of the wards, medications, or disease of concern, with an incidence of 33.3%–99.4% (Tefera et al., 2020). A study performed in the surgical department of a major city in southwest China reported that a total of 19.6% had at least one DTP (Qu et al., 2019). Age category, sex, presence of multiple comorbidities, rural residency, lower educational and economic status, functional dependence, impaired cognition, lower patient awareness, prolonged hospital stay, and polypharmacy were mentioned as determinants for DTPs in several studies (Ayalew et al., 2019; Short et al., 2023; Dammalapati et al., 2018). In Ethiopia, a study conducted at Jimma Medical Center among surgical patients revealed that 76% of the 300 study participants had a total of 449 DTPs identified, equating to an average of 1.97 per patient. The most prevalent types of DTPs were dose-too-low (27.6%) and dose-too-high (18.0%). The only independent predictors of DTPs were polypharmacy and hospital stays greater than 20 days (Tefera et al., 2020).

Limited data exist on the magnitude of DTPs and associated factors among adult surgical patients in Ethiopia, particularly in the current study setting. The prior study conducted at Jimma Medical Center did not consider variables such as preoperative complications like perforated appendicitis, peritonitis, gangrene, and types of anesthesia (local or general) used, which might have an impact on DTPs among surgical patients. Therefore, this study aimed to assess the magnitude of drug therapy problems and their associated factors among surgical patients admitted to the surgical ward of WUCSH, Hossana, Ethiopia.

This study will provide important information to tertiary hospitals, including study settings and healthcare providers about the magnitude of the drug therapy problem and its associated factors among adult surgical patients. The findings can be used to develop interventions and strategies to improve patient care by establishing protocols and guidelines for monitoring and managing DTPs in surgical patients. The findings may also be used as a baseline for researchers as well as for other stakeholders to reduce the burden of DTPs and their complications.

## Methods and materials

### Study area and study period

The study was conducted at the surgical ward of WUCSH in Hossana, central Ethiopia, from January 1 to 30 March 2024. Hosanna town is located at a distance of 232 km southwest of the capital city of Ethiopia, Addis Ababa, and 194 km northwest of Hawassa City. WUCSH was established in 1984 E.C.; and currently, the hospital is under the administration of Wachemo University. It provides healthcare services to more than 3.2 million people around Hosanna and nearby towns. It delivers different healthcare services to the community like internal medicine, surgery, gynecology and obstetrics, pediatrics, maternal and child health (MCH), tuberculosis (TB) and HIV (TB/HIV), intensive medical care, mental healthcare, dermatology and venereal disease services, pharmacy, and different laboratory services.

### Study design

A hospital-based cross-sectional study was conducted.

### Population

The source population was all adult patients admitted to the WUCSH surgical ward. The study population consisted of all adult patients admitted to the surgical ward of WUCSH from January 1 to 30 March 2024, and who fulfilled the inclusion criteria.

### Eligibility criteria

Adult patients admitted to the surgical ward, patients who were aged ≥18 years admitted for surgical procedures, and those who were hospitalized for >24 h were included in our study. However, those who have been readmitted within the data collection period, self-discharged, and severely ill patients were excluded from this study.

### Sample size determination and sampling technique

The sample size was calculated based on a single population proportion formula by taking the estimated prevalence of DTPs (76%) from the Jimma Medical Centre among adult surgical patients in a tertiary hospital (Tefera et al., 2020) as follows:

n=Zα22p1−pd2=∼ 281

Where n is the sample size required; p is the estimated prevalence of DTPs; Zα/2 is the standard normal deviation at a 95% confidence interval corresponding to 1.96; and d is the absolute error between the estimated and true population prevalence of DTPs of 5%. The sample sizes for associated factors were determined by considering different associated factors. By using EPI Info version 7 statistical software with the assumptions of confidence level 95%, power 80%, and the ratio of exposed to unexposed 1:1, the sample size was 110 surgical patients for polypharmacy and 111 for prolonged length of hospital stay. Therefore, the larger final sample size was 310 admitted surgical patients after adding a 10% non-response rate. A convenient sampling technique was used for this study.

### Data collection methods

A structured questionnaire was developed from reviewing different literature (English version) (Mohammed et al., 2022) and (Tefera et al., 2020). The questionnaire contains four sections: part I (patient-related factors), part II (disease-related factors), part III (medication-related factors), and part IV (drug therapy problems). Two trained pharmacists and one nurse professional participated as data collectors in data collection. Two days of training were given to the data collectors and one senior clinical pharmacist to familiarize them with the data collection instrument as well as how to collect the necessary data from charts and how to conduct patient interviews. Daily supervision and routine follow-up were done by the principal investigator, and drug therapy problems were identified by a multidisciplinary team which includes senior clinical pharmacists.

Relevant information about each patient, like patient characteristics, laboratory results, current medications, co-morbidities, length of hospitalization, and relevant previous medical and medication histories, was recorded using data abstraction format by reviewing patients’ medical charts. Additional information and clarifications on some patient’s medical information were obtained through interviewing the patients. Micromedex^®^ and Lexicomp drug interaction checker were used to identify drug-drug interactions. Identified DTPs were recorded and classified using the DTP registration format taken from Cipolle’s model, which has details of DTP measurement and classifications (Cipolle et al., 2012b).

### Study variables

Dependent variable: Drug therapy problems

#### Independent variables

Patient-related factors: Age, sex, religion, occupation, education status, monthly income, place of residency, marital status, alcohol consumption, khat chewing, and smoking status.

Disease-related (clinical) factors: Presence of comorbid conditions, number of comorbid conditions, presence of complications, surgical procedure done, types of procedure, hospitalization since the last year and length of hospitalization for current conditions.

Drug-related factors: types of medications, number of medications, preadmission medication, types of anesthesia used, postoperative drugs, postoperative pain management, patient drug compliance, prescribing preference based on guidelines, and number of drugs used at a time.

#### Operational definition

Drug therapy problem: an event or circumstance involving drug therapy that actually or potentially interferes with desired health outcomes in this study patient is considered to have DTP if the patient has one or more of the following DTP: dosage too low, dosage too high, need for additional drug therapy, ineffective drug, unnecessary drug, adverse drug reaction, and noncompliance (Cipolle et al., 2012b).

Adverse drug reaction: any noxious, unintended, and undesired effect of a drug, which occurs at doses used in humans for prophylaxis, diagnosis or therapy (Bekele et al., 2021).

Unnecessary drug therapy: The drug therapy is unnecessary because the patient does not have a clinical indication at this time.

Needs additional drug therapy: Additional drug therapy is required to treat or prevent a medical condition or illness from developing (Cipolle et al., 2012b).

Dosage too high: is a DTP where the dose is too high the dosing frequency is too short or the duration of therapy is too long for the patient (Cipolle et al., 2012b).

Dosage too low: is a DTP that occurs when the dose is too low to produce the desired outcome, the dosage interval is too long or the duration of therapy is too short (Cipolle et al., 2012b).

Ineffective drug therapy: The drug product is not effective at producing the desired response or outcome (Cipolle et al., 2012b).

Noncompliance: is a DTP that occurs when the patient does not understand the instructions or the patient prefers not to take or forgets to take the medication the cost of the drug product is not affordable for the patient, or the patient cannot swallow or self-administer the medication properly, or the drug product is not available for the patients (Cipolle et al., 2012b).

Polypharmacy: polypharmacy is the use of at least five drugs concurrently. Different strengths of the same drug were counted as one item. However, formulations of one drug requiring different routes of administration were regarded as separate items (Pazan and Wehling, 2021).

### Data quality control

A pre-test study was conducted among 5% of the calculated sample size (16 patients) before the days of the data collection period at Gembichu Primary Hospital to check for the uniformity and understandability of the questionnaire. Two days of training were given for data collectors with continuous supervision and follow-up during the data collection process. Every day, the principal investigator cross-checks the accuracy and consistency of the data. Data were stored in a safe and secure place, and all materials utilized for data gathering were organized sequentially.

### Methods of data analysis

The collected data was checked for its completeness, coded, and entered into Epi data version 3.1 and it was analyzed by using SPSS version 20 software. Descriptive statistics were performed to describe the demographic profile of the study participants. Cleaning was performed to check for frequencies, accuracy, consistencies, and missed values and variables. Binary and multivariable logistic regression was used to assess the association between independent variables and a dependent variable. A variable with a p-value less than 0.25 in the bivariable analysis was entered into the multivariable model. In multivariable logistic regression, model goodness of fit was checked by the Hosmer and Lemeshow test, and Multicollinearity was detected by variance inflation factor (VIF). Then, the adjusted odds ratio (AOR) along with a 95% CI was used to determine the strength of association with the level of statistical significance at a p-value less than 0.05. Finally, statistically significant factors were reported as factors associated with drug therapy problems for admitted surgical patients in this study.

## Results

### Sociodemographic and clinical data of study participants

During the 3-month study period, a total of 330 patients were admitted to the surgical ward. After excluding 26 patients, 304 patients fulfilled the eligibility criteria, and they were enrolled in our study. Among the study participants, 216 surgical patients were males, and more than one-third of study participants were in the age range of 31–50 years with a mean age of 44.41 (±17.39) years. More than half of the study participants were married, 161 (53.0%). About 106 (34.9%) had not attended formal education, and 141 (46.4%) of the study participants were farmers with a monthly income of less than 1000 ETB. Of the total patients, 119 (39.1%) had been hospitalized once since last year and the majority of them, 179 (58.9%), stayed at the hospital longer than 7 days. More than half of the study participants, 215 (70.7%), took more than five drugs (Table 1).

**TABLE 1:** Sociodemographic and clinical characteristics of hospitalized adult patients in surgical of WUCSH from January 1 to 30 March 2024 (n = 304).

Variables	Category	Frequency (%)
Sex	Male	216 (71.1)
	Female	88 (28.9)
Age	18–30 years	91 (29.9)
	31–50 years	111 (36.5)
	51–65 years	60 (19.7)
	Above 65 years	42 (13.8)
Marital status	Single	64 (21.1)
	Married	161 (53.0)
	Divorced	37 (12.2)
	Widowed	42 (13.8)
Religion	Protestant	143 (47.0)
	Orthodox	86 (28.3)
	Muslim	62 (20.4)
	Others*	13 (4.3)
Educational status	Cannot read and write	94 (30.9)
	Non-formal education	106 (34.9)
	Primary education (1–8 grade)	44 (14.5)
	Secondary education 9–12 grade	31 (10.2)
	Diploma and above	29 (9.5)
Occupation	Farmer	141 (46.4)
	Merchant	61 (20.1)
	Student	31 (10.2)
	Employer	26 (8.6)
	Housewife	40 (13.2)
	Others**	5 (1.6)
Monthly income in Ethiopian birr (ETB) #	Less than 1000 ETB	155 (51)
	1000–3000 ETB	63 (20.7)
	3001–5000 ETB	58 (19.1)
	Greater than 5000 ETB	28 (9.2)
Alcohol consumption	Yes	51 (16.8)
	No	253 (83.2)
Smoking status	Yes	21 (6.9)
	No	283 (93.1)
Khat chewing	Yes	47 (15.5)
	No	257 (84.5)
Place of Residency	Rural	195 (64.1)
	Urban	109 (35.9)
Hospitalization since last year	Never	86 (28.3)
	One time	119 (39.1)
	Twice	60 (19.7)
	≥ Three	39 (12.8)
Length of hospital stay in days	< Seven	125 (41.1)
	≥ Seven	179 (58.9)
Number of drugs taken	< Five	89 (29.3)
	≥ Five	215 (70.7)

*Catholic, Adventist, **Religious teachers’ Servants, #Classified after reviewing different literatures.

### Types of procedures performed

**FIGURE 1:**
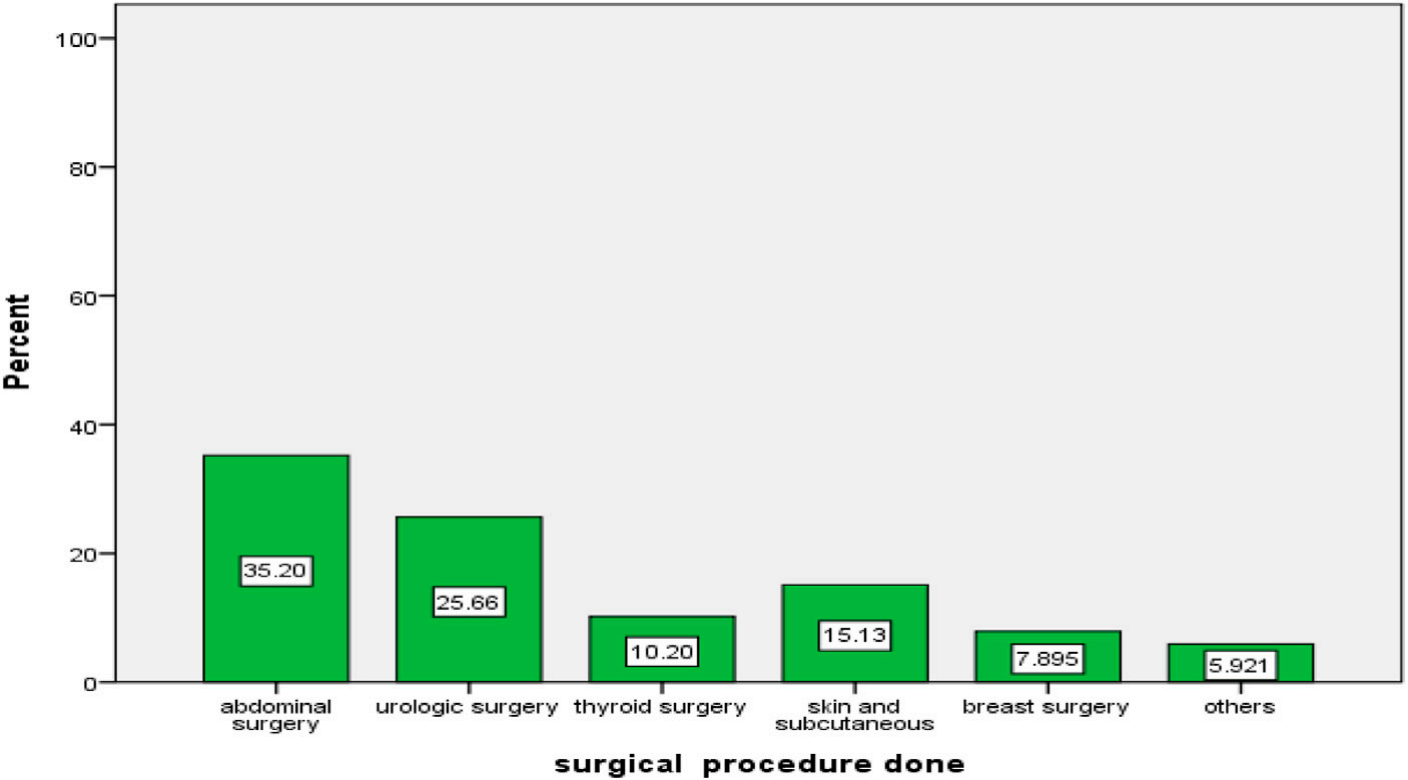
Types of surgery done during the study period in the surgical ward of WUCSH from January 1 to 30 March 2024 (n = 304).

**FIGURE 2:**
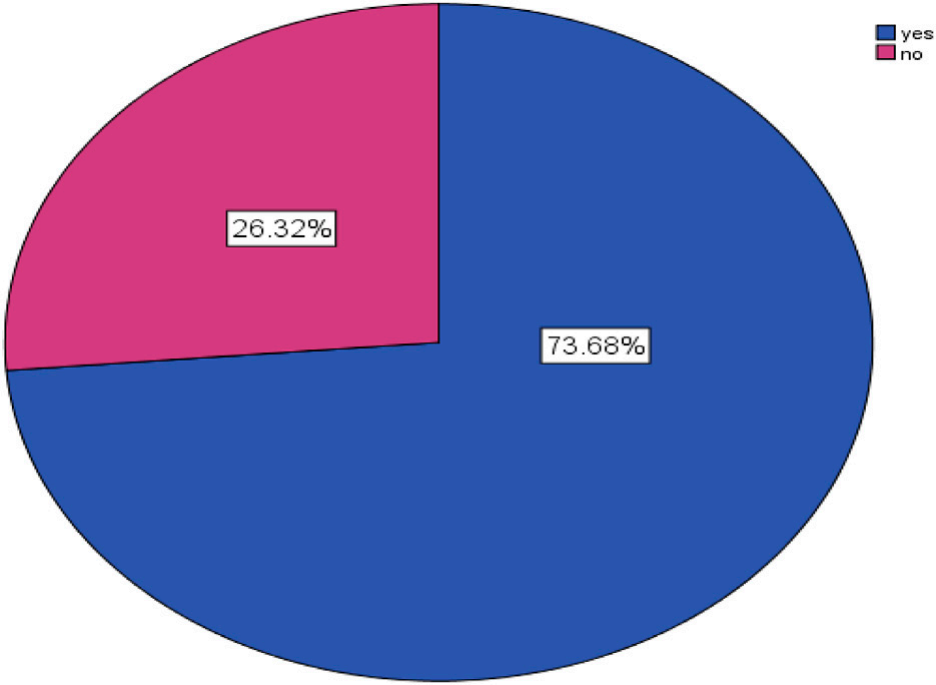
Magnitude of DTPs among study participants in the surgical ward of WUCSH from January 1 to 30 March 2024 (n = 304).

As demonstrated in Figure 2, the most common type of surgery done during the study period was abdominal surgery, 107 (35.2%). The remaining surgical procedures were urologic surgery 78 (25.7%), thyroid surgery 31 (10.2%), skin and subcutaneous 46 (15.1%), breast surgery 24 (7.9%), and others (5.9%) such as hernias, vascular, and esophageal surgery (Figure 1).

### Prescribed preadmission and surgical-related medications

Around 279 (91.7%) study participants had at least one preadmission medication prescribed for medical and surgical comorbidities. The most prescribed drug classes during the study period were anti-infective 90 (32.26%); cardiovascular drugs 42 (15.1%); anti-diabetic drugs 25 (9%); anti-thyroid drugs 40 (14.3%); alpha-1 antagonist drugs 27 (9.7%); respiratory drugs 18 (6.5%; and other drugs [tamoxifen, antiretroviral treatment, phenytoin, pyridoxine, amitriptyline, phenobarbital, and sodium valproate], 37 (13.3%) (Table 2).

**TABLE 2:** Description of used medications among adult surgical patients admitted at WUCSH from January 1 to 30 March 2024 (n = 304).

Types of drugs	Name of drugs	Frequency (%)
Preadmission medications	Anti-infective	90 (32.26%)
	Cardiovascular drugs	42 (15.1%)
	Anti-diabetic drugs	25 (9%)
	Anti-thyroid drugs	40 (14.3%)
	Alpha-1 antagonist drugs	27 (9.7%)
	Respiratory drugs	18 (6.5%)
	Other drugs*	37 (13.3%)
Preoperative medications	Ceftriaxone alone	146 (48)
	Ceftriaxone with metronidazole	77 (25.3)
	Ciprofloxacillin	62 (20.4)
	Other drugs**	19 (6.3)
Postoperative medications	Ceftriaxone alone	96 (31.6)
	Ceftriaxone with metronidazole	109 (35.9)
	Ciprofloxcacillin	59 (19.4)
	Other drugs***	40 (13.2)
Postoperative pain management	Tramadol alone	97 (34.6)
	Diclofenac with tramadol	99 (35.4)
	Diclofenac alone	37 (13.2)
	Other drugs****	47 (16.8)

*tamoxifen, antiretroviral treatment, phenytoin, pyridoxine, amitriptyline, phenobarbital, sodium valproate; **ampicillin; ***vancomycin, ceftazidime, meropenems heparin, steroid, and albumin; ****morphine, ibuprofen and paracetamol.

During the study period, ceftriaxone 146 (48%) and metronidazole with ceftriaxone 109 (35.9%) were the most frequently prescribed medications for the treatment of preoperative and postoperative surgical conditions, respectively. More than ninety percent of patients, 280 (92.1%), were provided postoperative pain management, diclofenac with tramadol 99 (35.4%) frequently prescribed (Table 2).

### The magnitude of DTPs and percentage of DTPs per patient

The magnitude of DTPs among study participants was found to be 73.68% (95% CI: 0.684–0.785) (Figure 2).

**FIGURE 3:**
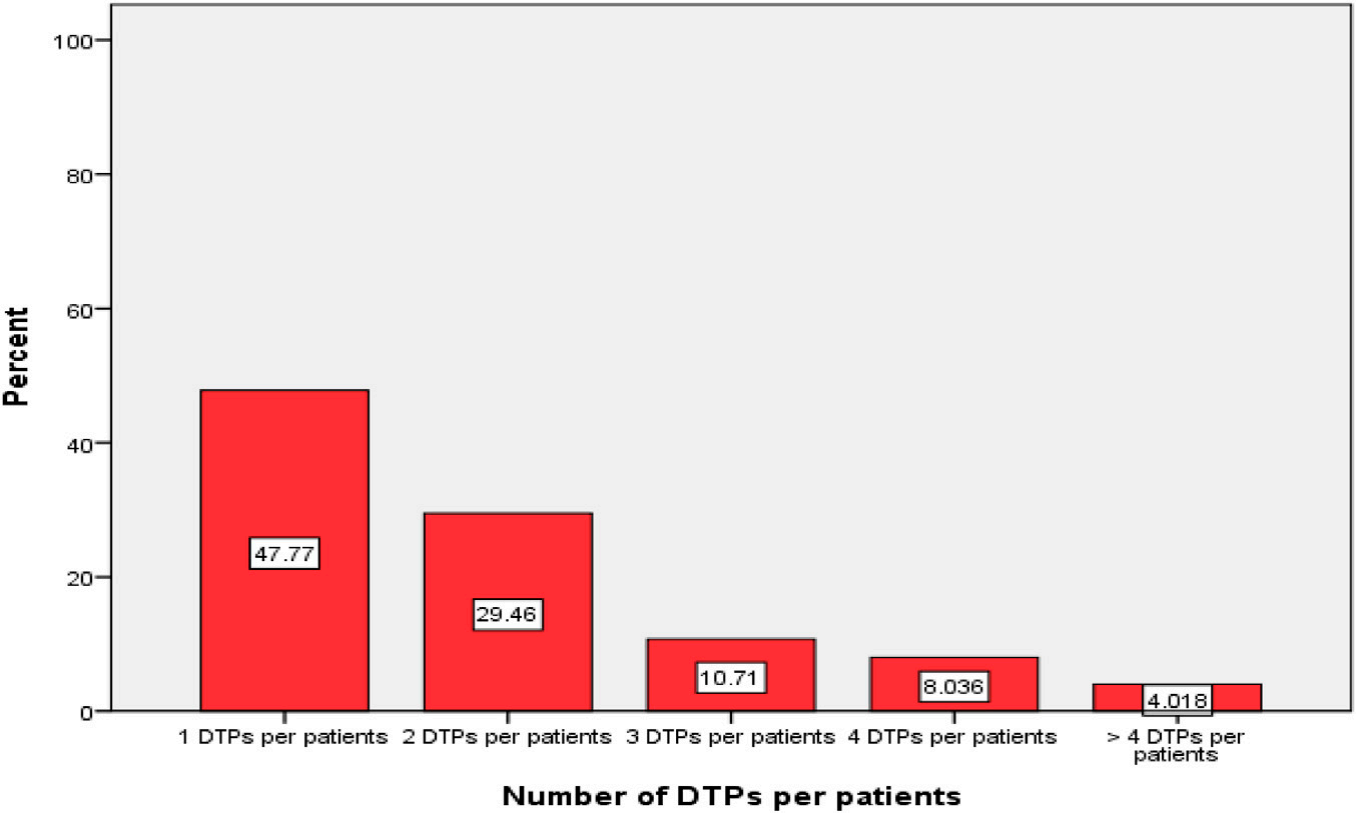
Percentage DTPs per-patients during the study period in the surgical ward of WUCSH from January 1 to 30 March 2024 (n = 304).

Among those study participants, 107 (47.77%) had one DTP, 66 (29.46%) had two DTPs, 24 (10.71%) had three DTPs, 18 (8.03%) had four DTPs and 9 (4.01%) of patients had greater than four DTPs in this study (Figure 3).

### Drug therapy problem types and their causes

Out of 304 study participants, a total of 464 DTPs were identified. Non-compliance was the most common (27%), followed by the need for additional drug therapy (21.1%). Other types included ineffective drug therapy, dosage too low, unnecessary drug therapy dosage too high, and adverse drug reactions (Table 3).

**TABLE 3:** Details of each identified DTP during the study period at the surgical ward of WUCSH from January 1 to 30 March 2024 (n = 304).

Description of DTPs		Numbers of DTPs	Total	Percentage
Unnecessary drug therapy	No valid medical indication	13	42	9.05%
	Multiple drug products are being used	12		
	Medical conditions more appropriately treated with non-drug therapy	6		
	To treat an avoidable adverse reaction associated with another medication	5		
	Drug abuse	6		
Need additional drug therapy	Required the initiation of the drug	50	98	21.1%
	Preventive drug therapy required	5		
	Required drug for synergistic or additive effects	43		
Ineffective drugs	The drug is not the most effective for the medical problem	23	71	15.3%
	Medical condition refractory to the drug product	23		
	The dosage form of the drug product inappropriate	12		
	Drug products are not effective in the indication	13		
Dosage too low	Dose too low	23	67	14.4%
	Dosage interval too infrequent	21		
	Drug interaction reduces the amount of active drug	11		
	Duration too short	12		
Adverse drug reaction	Drug products cause an undesirable reaction	6	24	5.17%
	Safer drug products are required due to risk	10		
	Drug interaction causes an undesirable reaction	8		
Dosage too high	Dose too high	11	37	8%
	Dosing frequency too short	14		
	Duration of drug therapy too long	12		
Non-compliance	Does not understand the instructions	22	125	27%
	Patients prefer not to take the medication	24		
	Forget to take the medication	24		
	Drug products are too expensive	23		
	The patient cannot swallow or self-administer	11		
	Drug product not available	10		
	Omission of the medication	11		
	Total		464	100

### Factors associated with drug therapy problems

#### Bivariate analysis

Variables such as educational status, types of surgery done, length of hospital stay, number of drugs prescribed, and postoperative use of antibiotics had a p-value of less than 0.25. These variables are taken in multivariable analysis. To identify factors associated with the occurrence of DTPs among study participants, the variables with a p-value of less than 0.25 in binary logistic regression were entered into multivariable logistic regression (Table 4).

**TABLE 4:** Univariate binary logistic regression analysis between explanatory and dependent variables among surgical patients at WUCSH from January 1 to 30 March 2024 (n = 304).

Variables	Category	DTPs		COR	(95% CI)	p-value
		Yes (%)	No (%)			
Sex	Male	160 (71.4)	56 (70)	1.071	0.613–1.874 1	0.809
	Female	64 (28.6)	24 (30)	0.35		
Age	18–30 years	70 (31.2)	21 (26.2)		1	0.733
	31–50 years	82 (36.6)	29 (36.2)	1.179	0.618–2.249	0.818
	51–64 years	44 (19.6)	16 (20)	1.212	0.572–2.571	0.678
	>65 years	28 (12.5)	14 (17.5)	1.667	0.745–3.731	0.582
Educational status	Cannot read and write	67 (29.9)	18 (22.5)		1	0.558
	Non-formal education	67 (29.9)	22 (27.7)	1.222	0.601–2.484	0.579
	Primary education (1–8 grade)	26 (11.6)	11 (13.8)	1.575	0.656–3.782	0.310
	Secondary education 9–12 grade	31 (13.8)	16 (20)	1.921	0.866–4.262	**0.108**
	Diploma and above	33 (14.7)	13 (16.2)	1.466	0.642–3.350	0.364
Types of procedures done	Abdominal surgery	83 (37)	29 (36.3)		1	0.261
	Urologic surgery	53 (23.6)	26 (32.5)	1.747	0.472–6.473	0.404
	Thyroid surgery	28 (12.5)	5 (2.2)	2.453	0.652–9.232	**0.185**
	Skin and subcutaneous	26 (11.6)	13 (16.2)	0.893	0.187–4.260	0.887
	Breast surgery	19 (8.4)	4 (5)	2.500	0.612–10.210	0.28
	Others#	15 (6.6)	3 (3.7)	0.429	0.204–5.442	0.951
Length of hospital stay	<Seven	81 (36.1)	44 (55)		1	**0.004**
	≥Seven	143 (63.8)	36 (45)	2.158	1.285–3.622	
Types of anaesthesia	General	160 (71.4)	61 (76.2)	1.284	0.711–2.319	0.407
	Regional	64 (28.6)	19 (23.6)	0.297		
Presence of Comorbidity	Yes	159 (71)	52 (65)	1.317	0.766–2.266 1	0.320
	No	65 (29)	28 (35)	0.327		
Presence of complications	Yes	90 (40.9)	29 (37.1)	1.170	1 0.687–1.991	0.564
	No	130 (59)	49 (62.8)	0.322		
Number of drugs taken per day	<Five	57 (25.4)	32 (40)		1 1.139–3.348	**0.015**
	≥Five	167 (73.2)	48 (60)	0.512		
Postoperative antibiotics use	No	49 (21.8)	65 (81.2)		1 8.123–29.486	**0.001**
	Yes	175 (78.1)	15 (18.8)	15.476		

Bold indicates the variable with a p-value of less than 0.25, COR, crude odds ratio, # Hernias, vascular surgery and esophageal.

#### Multivariable analysis

The multivariable logistic regression analysis showed that length of hospital stay, number of drugs taken per day, and administration of postoperative antibiotics had a significant association with the occurrence of DTPs. Accordingly, study participants who had stayed more than 7 days in the hospital were nearly 2.5 times to experience DTPs (AOR = 2.470, 95% CI: 1.243–4.909, p = 0.01) as compared to study participants who stayed less than 7 days in the hospital. The study participants who took more than five drugs per day were 2.87 times to experience DTPs (AOR = 2.874, 95% CI: 1.411–5.851, p = 0.004) as compared to study subjects who took less than five drugs per day. Whereas patients who took postoperative antibiotics were less likely to experience DTPs (AOR = 0.057, 95% CI: 0.028–0.115, p = 0.001) compared to those who did not take post-op antibiotics (Table 5).

**TABLE 5:** Multivariable binary logistic regression analysis for factors associated with DTPs during the study period at the surgical ward of WUCSH, 2024 (n = 304).

Variables	Category	DTPs		AOR (95% CI)	p-value
		Yes (%)	No (%)		
Educational status	Can’t read and write	67 (29.9)	18 (22.5)	1	0.457
	Non-formal	67 (29.9)	22 (27.5)	0.789 (0.275–2.262)	0.659
	Education primary education	26 (11.6)	11 (13.7)	1.184 (0.431–3.253)	0.743
	Secondary education	31 (13.8)	16 (20.0)	0.911 (0.272–3.052)	0.879
	Diploma and above)	33 (14.7)	13 (16.2)	2.037 (0.671–6.182)	0.209
Length of hospital stay	< Seven days	81 (36.1)	44 (55)	1	**0.01**
	≥ Seven days	143 (63.8)	36 (45)	2.470 (1.243–4.909)	
Surgery done	Abdominal surgery	80 (35.7)	27 (33.3)	1	0.393
	Urologic surgery	52 (23.2)	26 (32.5)	3.636 (0.7381–7.920)	0.113
	Thyroid surgery	26 (11.6)	5 (6.2)	4.562 (0.909–22.893)	0.065
	Skin and subcutaneous	32 (14.2)	14 (17.5)	1.705 (0.263–11.041)	0.575
	Breast surgery	19 (8.4)	5 (6.2)	3.766 (0.704–20.147)	0.121
	Others#	15 (6.6)	3 (3.70	2.319 (0.337–15.941)	0.393
Number of drugs taken	< Five drugs	57 (25.4)	32 (40)	1	**0.004**
	≥ Five drugs	167 (73.2)	48 (60)	2.874 (1.411–5.851)	
Postoperative antibiotics	No	49 (21.8)	65 (81.2)	1	**0.001**
	Yes	175 (78.1)	15 (18.8)	0.057 (0.028–0.115)	

Bold indicates the variable is statistically significant (95%, C.I), AOR, adjusted odds ratio; CI, Confidence Interval. #Hernias, vascular and esophageal surgery.

## Discussion

The use of medication therapy is expanding, which makes it more difficult to prescribe medicines appropriately, and suboptimal use of medication is preventable (Levy, 2023). It has been demonstrated that handling DTPs using pharmaceutical care services can result in financial savings, a decrease in adverse drug events, a decrease in mortality, and an improvement in overall quality of life (Kjeldsen et al.). Assessing the extent of DTPs and factors associated with this problem among hospitalized patients is very crucial for the prevention and control of DTPs to achieve positive therapeutic outcomes in hospitals (Oki et al., 2021). In this study, the magnitude of DTPs was high, and factors associated with DTPs among adult admitted surgical patients in the study setting were prolonged length of hospital stay, number of drugs taken per day, and administration of postoperative antibiotics.

The current study showed that nearly three-fourths of study participants (73.68%) admitted to the surgical ward of WUCSH during the study period were experienced DTPs. This result is higher than a study conducted in Canadian tertiary care hospitals (66.4%) (Neville et al., 2014). However, the prevalence of DTPs is lower than the current finding in tertiary hospitals of South India (Alagiriswami et al., 2009), and Southwest China (Qu et al., 2019), which reported 7.9% and 42.8%, respectively. These differences might be due to study settings, study design, sampling technique, and healthcare professionals’ skill variation. This result is consistent with a study conducted in southwest Ethiopia, which showed a prevalence of 69.5% (Mohammed et al., 2022). The present study also showed a similar prevalence of DTPs with another study conducted in Malaysia (76.1%) (Zaman Huri et al., 2014).

In this study, a total of 464 DTPs were identified, with approximately 47.77% (95% CI: 0.41–0.55) of participants having at least one DTP. This finding is similar to a study conducted at tertiary and academic medical centers (48%) in Northwestern Chicago (Phatak et al., 2016) and tertiary hospitals in Chongqing (55.4%), Southwest China (Qu et al., 2019); and lower prevalence (60%) in New Zealand (Wood and Lightfoot, 2018). These discrepancies highlight how different healthcare practices, resources, and patient management approaches across various countries and facilities can impact the prevalence of DTPs in tertiary hospitals.

Non-compliance was the most frequently identified DTP, accounting for 27% (95% CI: 0.23–0.31) in the current study. This finding is slightly lower than the non-compliance rate of (37.8%), reported studies involving surgical patients in New Zealand (Wood and Lightfoot, 2018). This discrepancy might result from differences in how non-compliance was identified, the resources available in the clinical settings, the sample size used, or the characteristics of the study population and differences in post-surgical care practices in different hospitals.

The current finding showed that need for the additional drug therapy was the second most commonly identified DTP with a prevalence of 21.1% (95% CI:0.176–0.25). This result is similar to the study conducted in Canada at adult tertiary care hospitals (25.5%) (Haley et al., 2009). However, the need for additional drug therapy was the major category of drug-related problems in referral hospitals in Kenya (Degu et al., 2023). The variation could be due to the level of pharmaceutical care experience and clinical practice which can help to easily identify and classify DTPs.

In the present study, approximately 15.3% (95% CI: 0.12–0.19) of DTPs were attributed to the prescribing of ineffective drugs. This finding was lower than the study conducted in Malaysian (45.9%) (Zaman Huri et al., 2014) and in China (25.1%) (Meng et al., 2021); and the higher than finding was reported from a teaching hospital in Amman, Jordan which showed that the ineffective drugs (6.5%) were the most frequent observed DTPs (AbuRuz et al., 2021). The discrepancy might be the factors such as different patient populations, sample size, and differences in healthcare practices between settings could have contributed to the higher detection of ineffective prescriptions.

In this study, the dosage too low was 14.4% (95% CI: 0.11–0.18). This finding is higher than a study accompanied in Canada (9%) and Saudi Arabian (11%) (Aljadhey et al., 2014). However, it is lower than the study done in Ethiopia which showed dosage too low was 27.6% (Tefera et al., 2020). This difference might be due to the study setting, study design, sample size, involvement of clinical pharmacist, and difference in patient understanding use of medications through different ways.

According to the present study, 13.17% of safety-related problems were observed among surgical patients, of which 8% were dosages too high and 5.17% were adverse drug reactions. This finding was lower than the study conducted in Jordan (20.2%) (AbuRuz et al., 2021); and higher than the study revealed in Canada (10.5%) (Meng et al., 2021). The difference might be due to the categorization and classification of DTPs, experience practice in detecting, and reporting of ADR, and study design.

The least prevalent DTP was unnecessary drug therapy, which accounts for 9.05%, often due to prescribing medication without a valid medical indication in this study. This finding is lower than the study conducted in China (16.8%) (Qu et al., 2019), and Jordan (18.9%) (AbuRuz et al., 2021). The variation could be due to the study design, study setting, study populations, and differences in healthcare practice. However, it is higher than the reported in a study conducted by Jimma Medical Center in Ethiopia that indicated 1.97% (Mohammed et al., 2022).

In the current study, the number of drugs taken per day, length of hospital stay, and post-operative antibiotics were shown to be associated factors for the occurrence of DTPs. Patients who took greater than or equal to five drugs per day were 2.874 times have DTPs compared to patients who took less than five drugs per day. This finding is in line with a study conducted at Jimma Medical Center (Tefera et al., 2020). This study indicates that polypharmacy predictor for the occurrence of DTPs and it is a generalizable risk factor in healthcare settings, which means that patients’ drug regimens need to be reviewed frequently, especially if they are prescribed five or more prescriptions (Al Hamid et al., 2014). Consequently, the use of clinical pharmacists in hospitalized patient care might help lower the risk of DTPs and minimize the hazards related to polypharmacy (Bhatty et al., 2018).

The patients who received antibiotics postoperatively decreased the occurrence of DTPs by 0.057 (AOR = 0.057, 95%CI: 0.028–0.115, p = 0.001) than those who didn’t take antibiotics postoperatively in this study. This finding is similar to a study done at Jimma Medical Center (Mohammed et al., 2022). This finding showed that postoperative antibiotics use is associated with a lower incidence of DTPs suggesting that antibiotics play a crucial role in preventing complications like surgical site infections that could lead to DTPs when appropriately prescribed. Therefore, reducing the likelihood of complications and subsequent need for additional medications which in turn lowers the risk of DTPs among surgical patients.

The present study showed that patients whose hospital stay was greater than 7 days were 2.5 times more likely to have DTPs than patients whose hospital stay was less than 7 days. This result was consistent with those studies done by Jimma Medical Center (Tefera et al., 2020). This could be due to prolonged hospitalization may increase the number of drugs, drug-drug interactions, reasons for changing medications, a chance to acquire new infections, and non-adherence which increases the possibility of the development of DTPs.

### Limitations of the study

The study’s strength was demonstrated through interviewing the participants who had accurate and updated information, which enabled easy identification of various DTP categories. Additionally, by including all adult surgical patients, both elective and emergency, the study was able to obtain an adequate study sample size. This study also had some limitations: first, since this study is a cross-sectional study, it cannot establish causal relationships. Second, the study was conducted at a single institution with convenience sampling, and this may affect the generalizability of the findings. Finally, this study did not consider severity and types of surgery performed, physician variability, impacts of each DTP on the patient’s outcomes, and DTPs were considered based on patients’ responses and/or from medical records without the establishment of a causal relationship. Therefore, the results of this study should be interpreted with caution, and we recommend further multicenter studies with follow-up to establish a causal relationship between the occurrence of DTPs and associated factors by addressing other limitations of this study.

## Conclusion

The present finding showed a high magnitude of DTPs among patients in the surgical ward at WUCSH, and this finding highlights the need for significant attention and intervention in this area. The most frequently identified DTP was noncompliance, indicating a critical area for improvement in patient adherence to prescribed therapies. Additionally, the study identified several factors associated with an increased likelihood of DTPs, including polypharmacy, prolonged hospital stay in days, and the use of postoperative antibiotics. Generally, this study emphasizes the importance of targeted strategies to minimize DTPs through clinical pharmacist integration, developing protocols for DTP screening, and patient counselling strategies to improve the treatment outcomes of surgical patients. Nationalwide interventional studies with multicenter settings will be needed to establish the impacts of DTPs on treatment outcomes of surgical patients in Ethiopia.

## Data Availability

The raw data supporting the conclusions of this article will be made available by the authors, without undue reservation.

## Abbreviations

ADEs, Adverse Drug Events; ADR, Adverse Drug Reaction; CVS, Cardiovascular System; DRHs, Drug-related hospitalizations; DTPs, Drug Therapy Problems; PCNE, Pharmaceutical Care Network Europe; US, United States; WHO, World Health Organization; and WUCSH, Wachamo University Comprehensive Specialized Hospital.
